# Psychiatric morbidity in children involved in bullying treated at the Free State Psychiatric Complex

**DOI:** 10.4102/sajpsychiatry.v29i0.2000

**Published:** 2023-03-30

**Authors:** Mosa Masakala, Matieho Mofokeng, Amanda Muchocho, Siphesihle Sibisi, Johan le Roux, Helene le Roux, Gina Joubert

**Affiliations:** 1 Department of Psychiatry, Faculty of Health Sciences, University of the Free State, Bloemfontein, South Africa; 2 Department of Biostatistics, Faculty of Health Sciences, University of the Free State, Bloemfontein, South Africa

**Keywords:** bullying, victims and bullies, psychiatric conditions, health profile, involved versus not involved

## Abstract

**Background:**

Bullying is a multifaceted problem with many consequences.

**Aim:**

This study aimed to determine the psychiatric morbidity of children involved in bullying, either as bullies or victims, treated at the Child and Adolescent Mental Health Care Centre of the Free State Psychiatric Complex (FSPC).

**Setting:**

Free State Psychiatric Complex, Bloemfontein, South Africa.

**Methods:**

This retrospective cross-sectional study included children under 18 years treated at the FSPC Care Centre between January and September 2017. Information was extracted from patient files.

**Results:**

Of 288 patients, 98 (34.0%) were involved in bullying: 66 were bullies, 28 victims, 3 bully-victims, and 1 unspecified. For gender and family structure, there were no statistically significant differences between children involved and those not involved in bullying and between bullies and victims. Almost all bullies (95.4%) had aggression as presenting complaint compared with 39.3% of the victims (*p* < 0.01). Statistically significantly more victims, than bullies, reported sadness (21.4%, 4.6%, *p* = 0.02). Attention deficit/hyperactivity disorder (ADHD) was diagnosed in most children, both involved (73.5%) and not involved (63.2%). Statistically significant differences for the presence of conduct disorder were found between children involved and those not involved in bullying (31.6%, 10.0%, *p* < 0.01) and between bullies and victims (39.4%, 14.3%, *p* = 0.02).

**Conclusion:**

The prevalence of conduct disorder diagnosis was more common in bullies than in victims and those involved in bullying as opposed to those not involved.

**Contribution:**

Psychiatric information of bullying victims and perpetrators in the Free State, which had a high prevalence of bullying in a national survey.

## Introduction

Bullying is a multifaceted and dynamic form of interpersonal aggression influenced by multiple factors.^1^ It involves purposeful acts of a single or group of perpetrators on a less powerful victim. These acts can be overt or covert and are not isolated but continuously repeated to hurt the other person involved.^2^ Bullying is not simply a problem between the victim and the bully but rather a phenomenon occurring in a social context manifested in different patterns of relationships with several consequences for all persons involved.^1^

A United Nations Educational, Scientific and Cultural Organization (UNESCO) publication^3^ recently illustrated the global prevalence of bullying victimisation. Findings showed that almost a third of children were bullied at least once during a 1-month period.^3^ Studies in South Africa also reported on the frequency of bullying behaviour and victimisation during childhood and adolescence. Boyes et al.^4^ conducted the first longitudinal study regarding bullying victimisation in South Africa, studying children and adolescents aged 10–17 years with follow-up after 1 year. More than half of the children and adolescents in this study reported being the victims of bullying. In a national study of South African school-going children aged 10–12 years investigating the prevalence of being hit, left out and called unkind names, the Free State province had the highest prevalence of children being hit by other children (33.3%).^5^ Nationally, the prevalence of being called unkind names was more than 30%.^5^ A study performed at Lentegeur Hospital Child and Adolescent Mental Health Service in the Western Cape, found that 56.7% of the sample of 13–18-year-olds were involved in cyberbullying: 6.2% as cyber-bullies, 20.6% as cyber-victims and 29.9% as bully-victims.^6^

Numerous studies have investigated individual and social contributing factors to bullying behaviour. Individual characteristics associated with bullying behaviour include, for example, age, gender, socio-economic status, ethnicity and special education needs.^7^ Familial influences with bullying and conduct problems in the relationship between the child and parent consisted of low maternal warmth, family dysfunction, low socio-economic status and the antisocial behaviour of the parents.^8^ Fink et al.^7^ investigated school-level predictors and found that characteristics such as school deprivation and poor climate (e.g. poor support and connectedness) to be predictive of bullying behaviour.

Children can be involved in bullying as either bullies (doing the bullying), victims (being abused or intimidated by a bully) or bully-victims (being both bully and victim). Males show a preponderance of being the bully and victim.^9,10^ Smith et al.^10^ reported on the consistency of gender differences by reviewing five cross-national databases. Findings supported the preponderance of males as perpetrator and victim, although victim rates were lower overall. This gender difference was also supported in South Africa, where Juan et al.^11^ and Manuel et al.^5^ similarly found males to be more likely to be the victims of bullying. Gender differences are also apparent in different types of bullying, where males are more likely to be involved in aggressive bullying, such as physical fighting and females more in relational bullying.^4,10^

Bullying is a traumatic experience that can result in serious consequences for the mental and physical health of those affected. Various bullying exposures have been associated with poor academic outcomes^12,13^ and poor physical and mental health.^4,14,15^ The association between somatic symptoms and bullying is evident in previous research.^16^ Victims of bullying frequently report somatic symptoms, such as headaches, weakness and pain associated with their stomach, chest, arms and legs.^17^ Victims of bullying are vulnerable to both internalising and externalising symptoms, such as anxiety, depression, post-traumatic stress and conduct problems.^4^ In addition, perpetrators are also associated with both externalising and internalising symptoms, such as conduct problems and a longitudinal association with depression.^8,18^ The systematic review by Serafini et al.^19^ found that bullies and victims were at increased risk of suicidality and non-suicidal self-injury. Armitage^20^ pointed out that bullies and victims have poor health and poor social and educational outcomes in childhood and adolescence, with adverse mental health outcomes most common in bully-victims.

It has been reported that mental health predisposed to being the victim of bullying, illustrating ‘bi-directionality of relationships between bullying victimisation and mental health outcomes’ (p. 1313).^4^ For example, Cuba Bustinza et al.^21^ reported on the characteristics of children diagnosed with attention-deficit/hyperactivity disorder (ADHD) using data from a national survey. Almost half of the children diagnosed with ADHD were victims (46.9%), compared with 16.2% who were perpetrators. The systematic review of Simmons and Antshel^22^ provided findings on the positive association between disorders such as ADHD, depression and bullying involvement. Children with a diagnosis such as ADHD might be more vulnerable to bullying involvement.

Arseneault^23^ highlighted the persistent effect of bullying and found that mental health problems, poorer physical health and economic hardship in adulthood are associated with childhood victimisation. Copeland et al.^24^ also reported on the adult psychiatric outcomes of children and adolescents involved in bullying. Elevated rates during adulthood were found for anxiety disorders (victims), depression (bullies and victims) and antisocial personality disorder (bullies).

Studies tend to focus on the victims of bullying. For example, the systematic review of Serafini et al.^19^ found that of the 29 studies that met their inclusion criteria, all included victims, four included bullies, and one included bully-victims. A study that compares bullies and victims of bullying could give valuable insight into those dealing with this type of behaviour.

## Aim

This study aimed to determine the psychiatric morbidity of children involved in bullying, either as bullies or victims, being treated at the Child and Adolescent Mental Health Care Centre of the Free State Psychiatric Complex (FSPC).

Specific objectives were to:

- determine the health profile (presenting physical and emotional complaints and clinical diagnoses), family structure and academic performance of children being treated at the FSPC who are involved in bullying
- compare these factors with those of children who are not involved in bullying
- compare these factors between bullies and victims.

## Research methods and design

A cross-sectional study was conducted in 2017 at the Child and Adolescent Mental Health Care Centre. This care centre is part of the FSPC and provides tertiary outpatient services to children and adolescents residing within the Free State province. The study population included all children under the age of 18 years who were treated at the care centre between January and September 2017. Interviews were conducted by different members of the multi-professional team and psychiatric diagnoses were confirmed during ward rounds. Academic performance was rated as good, average or poor by the professionals conducting the assessment interview based on information provided by the parent or guardian or child.

### Data collection

Data were collected from the patient files by means of a structured data form designed by the researchers. Information included demographic characteristics, involvement in bullying, family structure, academic performance and psychiatric morbidity (physical and referral complaints, clinical diagnosis).

### Pilot study

A pilot study was conducted on the first five files of children from the study population. The structured data form was confirmed to be adequate for the purpose of this study. No changes were made to the data form and the collection steps were not amended. Data from these files were included in the main study.

### Data analysis

The data were entered into an Excel spreadsheet. The analysis was carried out by the Department of Biostatistics, Faculty of Health Sciences, University of the Free State (UFS). Results are summarised by frequencies and percentages (categorical variables) and medians and interquartile ranges (numeric variables because of skew distributions). Subgroups were compared using Mann–Whitney tests (numerical variables) and chi-squared or Fisher’s exact tests (categorical variables, depending on cell sizes).

### Ethical considerations

The study was approved by the UFS Health Sciences Research Ethics Committee [UFS-HSD2017/0620], the Free State Department of Health, and the Ethics Committee of the FSPC. Ministerial consent for non-therapeutic research on minors was obtained. Each file was given a unique study number to ensure confidentiality.

## Results

**FIGURE 1:**
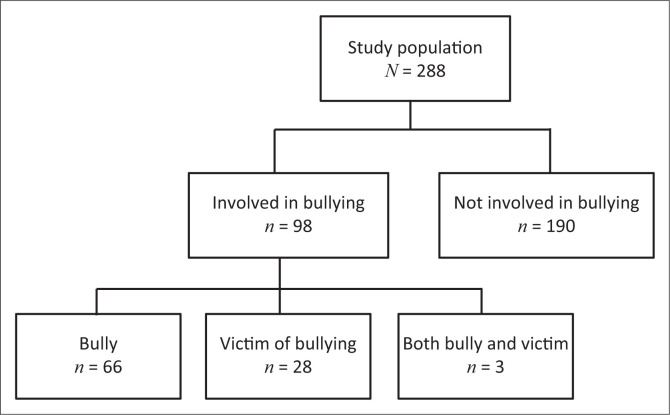
Involvement of children in bullying.

As shown in Figure 1, 288 patient files were reviewed, and 98 (34.0%) children were identified as being involved in bullying, either as bully (*n* = 66; 22.9%), victim (*n* = 28; 9.7%), or bully-victim (*n* = 3; 1.3%). One file indicated that the child was involved in bullying but did not specify the type of involvement.

The median age of children involved in bullying was 9 years (range 4–17 years), while children not involved in bullying had a median age of 11 years (range 2–17 years). The median ages of bullies and victims were 9 years.

More than 70% of children involved and those not involved in bullying were male (Table 1). A larger percentage of the bullies (78.8%) compared with the victims (67.9%) were male. The highest percentage of children involved in bullying (45.9%) lived with both parents, while 42.3% of the children not involved lived with a single parent. For gender and family structure, there were no statistically significant differences between the children involved and those not involved in bullying and between the bullies and the victims.

**TABLE 1:** Demographic details of children treated at the Free Sate Psychiatry Complex.

Variable	Bullies			Victims			Involved in bullying†			Not involved in bullying		
	*N*	*n*	%	*N*	*n*	%	*N*	*n*	%	*N*	*n*	%
**Gender**												
Male	66	52	78.8	28	19	67.9	98	74	75.5	190	136	71.6
Female	66	14	21.2	28	9	32.1	98	24	24.5	190	54	28.4
**Family structure**												
Both parents	66	31	47.0	28	13	46.4	98	45	45.9	189	66	34.9
Single parents	66	19	28.8	28	11	39.3	98	31	31.6	189	80	42.3
Guardian	66	8	12.1	28	2	7.1	98	12	12.2	189	27	14.3
Other	66	8	12.1	28	2	7.1	98	10	10.2	189	16	8.5
**Academic performance**												
Good	53	4	7.6	24	1	4.2	81	5	6.2	176	9	5.1
Average	53	12	22.6	24	12	50.0	81	26	32.1	176	63	35.8
Poor	53	37	69.8	24	11	45.8	81	50	61.7	176	104	59.1

† , This group includes the three children who were involved as both bully and victim and the one child where the involvement was not specified.

Children involved (61.7%) and those not involved (59.1%) in bullying mostly showed poor academic performance. Half (50.0%) of the victims had average academic performance compared with 22.6% of the bullies (*p* = 0.06).

Headache was the most frequently reported physical complaint among those involved in bullying (12.2%) as well as those who were not (12.1%) (Table 2). Victims (17.9%) reported a higher percentage of headaches than bullies (10.6%). There were, however, no statistically significant differences between any of the groups concerning physical complaints.

**TABLE 2:** Physical and referral complaints of children involved and not involved in bullying.

Complaints	Bullies *N* = 66		Victims *N* = 28		Involved in bullying‡ *N* = 98		Not involved in bullying *N* = 190	
		
	*n*	%	*n*	%	*n*	%	*n*	%
**Physical complaints†**								
Headache	7	10.6	5	17.9	12	12.2	23	12.1
Abdominal pain	3§	4.6	0	0.0	3§	3.1	11	5.8
Fatigue	0	0.0	2	7.1	2	2.0	2	1.1
Dizziness	0	0.0	1	3.6	1	1.0	3	1.6
Other	6	9.1	0	0.0	7	7.1	14	7.4
**Referral complaints†**								
Aggression	62§	95.4	11	39.3	77§	79.4	83	43.7
Social withdrawal	18	27.3	8	28.6	26	26.5	42	22.1
Anxiety symptoms	2	3.0	3	10.7	5	5.1	19	10.0
Depressive symptoms	1	1.5	3	10.7	4	4.1	22	11.6
Sadness	3	4.6	6	21.4	9	9.2	15	7.9
Irritability	10	15.2	7	25.0	18	18.4	27	14.2
Other	2	3.0	3	10.7	5	5.1	14	7.4

† , More than one option could be selected.

‡ , This group includes the three children who were involved as both bully and victim and the one child where the involvement was not specified.

§ , *n* = 1 missing.

Children involved in bullying were statistically significantly more likely to present with aggression than children not involved (*p* < 0.01). Almost all bullies (95.4%) presented with aggression compared with 39.3% of the victims (*p* < 0.01). Social withdrawal was the second most common referral complaint for bullies (27.3%) and victims (28.6%). There was a statistically significant difference between children involved in bullying (4.1%) versus those not involved (11.6%) about depressive symptoms (*p* = 0.04). Statistically significantly more victims than bullies reported sadness (*p* = 0.02).

The clinical diagnoses of the children are summarised in Table 3. A child could be diagnosed with more than one clinical disorder. Attention deficit/hyperactivity disorder was diagnosed in most children, both involved (73.5%) and not involved (63.2%) in bullying. Statistically significant differences for the presence of conduct disorder were found between children involved and those not involved in bullying (*p* < 0.01) and between bullies and victims (*p* = 0.02). Statistically significantly more children involved in bullying than those not involved were diagnosed with optional defiant disorder (*p* = 0.01). A higher percentage of bullies (33.3%) were diagnosed with oppositional defiant disorder than victims (21.4%); however, the difference was not statistically significant.

**TABLE 3:** Clinical diagnoses of children involved and not involved in bullying.

Clinical diagnoses†	Bullies *N* = 66		Victims *N* = 28		Involved in bullying‡ *N* = 98		Not involved in bullying *N* = 190	
	*n*	%	*n*	%	*n*	%	*n*	%
**1. Neurodevelopmental disorders**								
Intellectual disability	5	7.6	2	7.1	8	8.2	10	5.3
Autism spectrum disorder	1	1.5	0§	0.0	2§	2.1	5	2.6
Attention deficit/hyperactivity disorder	49	74.2	20	71.4	72	73.5	120	63.2
Specific learning disorders	10	15.2	2	7.1	13	13.3	30	15.8
**2. Psychotic disorder**	0	0.0	1	3.6	1	1.0	4	2.1
**3. Depressive disorders**	9	13.6	1	3.6	10	10.2	16§	8.5
**4. Anxiety disorders**								
Separation anxiety disorder	4	6.1	2	7.1	6	6.1	6	3.2
Generalised anxiety disorder	3	4.6	4	14.3	7	7.1	12	6.3
**5. Obsessive compulsive disorder**	1	1.5	1	3.6	2	2.0	2	1.1
**6. Trauma and stressor-related disorders**								
Post-traumatic stress disorder	3	4.6	0	0.0	3	3.1	6	3.2
Adjustment disorder	3	4.6	0	0.0	3	3.1	3	1.6
**7. Feeding and eating disorders**								
Anorexia nervosa	0	0.0	2	7.1	2	2.0	6	3.2
Eating disorders (other)	2	3.0	1	3.6	3	3.1	1	0.5
**8. Elimination disorder**	8	12.1	0	0.0	8	8.2	22	11.6
**9. Disruptive, impulse-control and conduct disorders**								
Oppositional defiant disorder	22	33.3	6	21.4	29	29.6	30	15.8
Conduct disorder	26	39.4	4	14.3	31	31.6	19	10.0
**10. Substance use disorder**	0	0.0	2	7.1	3	3.1	7	3.7
**11. Other**	0	0.0	1	3.6	1	1.0	5	2.6

† , More than one option could be selected.

‡ , This group includes the three children who were involved as both bully and victim and the one child where the involvement was not specified.

§ , *n* = 1 missing.

## Discussion

This study explored the psychiatric morbidity in children involved in bullying treated at a child and adolescent psychiatric outpatient centre. Approximately a third of the children treated at the centre during the study period were involved in bullying, mainly as bullies. Paruk and Nassen^6^ found that more than half of children aged 12–18 at a similar institution were involved in cyberbullying, mainly as bully-victims. Percentages of children involved in bullying may be lower because of the wider age group included and because cyber activities might have been less common at the time of our study.

Children in the study were predominantly male. Males are more prone to be bullies and victims.^9,10^ In this study, the percentage of males was slightly higher in the bullies than in the victims. As found in the study by Smith et al.,^10^ males represented the majority as the perpetrator and victim of bullying, with the victim showing a smaller representation. The predominance of males as victims is also reflected in previous South African studies.^5,11^ Fink et al.^7^ found individual characteristics, such as being male and of poor socio-economic status to be more likely to engage in bullying.

An association between family structure and involvement in bullying was not apparent in this study, with a greater percentage of children involved in bullying living with both parents, and most children not involved in bullying living with a single parent. Other factors that were not observed, such as family dysfunction and instability, might have provided different results, as was found in the study of Ganesan et al.^8^ Poor academic performance was a common factor among all children in this study group, indicating a possible relation between academic performance and mental health dysfunction.

Only a few physical complaints were reported by bullies and victims, such as fatigue, headaches, dizziness and abdominal pain. This finding was not expected as the literature indicated that a large number of physical complaints occur in these groups.^14,16,17^ Clearer patterns were observed regarding referral complaints, with nearly all bullies presenting with aggression. This finding might be explained by the predominantly male representation of the study population, as aggression and gender show an association. Males tend to be more directly involved in bullying behaviour, such as physical aggression, whereas females tend to act more indirectly through relational means.^4,10^ However, there was no significant difference between bullies and victims in terms of social withdrawal, contrary to studies showing that victims are prone to social withdrawal.^25^ Sadness, depressive symptoms and irritability were more frequent in victims than in bullies.

In both bullies and victims, ADHD was a common diagnosis, with a higher percentage in both groups than in children not involved in bullying. Previous research shows a positive association between ADHD and bullying involvement. Cuba Bustinza et al.^21^ reported a higher prevalence of victimisation in children diagnosed with ADHD. This study showed no statistically significant difference between bullies and victims. Depressive disorders, conduct disorder, oppositional defiance and adjustment disorder were more prominent in bullies, aligning with previous research indicating that bullying behaviour is associated with externalising and internalising symptoms.^8,18^ Only conduct disorder was statistically significantly higher. This finding was expected as bullying is a diagnostic criterion for conduct disorder.^26^ Conduct disorder is associated with aggressive behaviour, which is more prominent in bullies and was the main presenting complaint of this group. Manuel et al.^5^ confirmed the high prevalence of physical aggression in the Free State province compared with other South African provinces.

An unexpected finding was the low prevalence of depressive disorders in the victim group, as research highlights the association between victimisation and mental health problems, such as depression and anxiety.^4,15^ However, statistically, more victims reported sadness, which might be indicative of the presence of internalising symptoms, although not sufficient to warrant a clinical diagnosis. Paruk and Nassen^6^ found no significant association between cyberbullying and psychiatric diagnoses.

## Study limitations

This study focused only on children seen at a psychiatric treatment facility and relied on information in patient files. If patients did not volunteer information or were not directly questioned on bullying involvement, the information would not have been observed. Details on the types of bullying, such as verbal, physical, relational and cyberbullying, were limited. The research on bullying involvement might be strengthened by focusing on a narrower age group and using tailored questionnaires with patients and families to provide more detailed results.

## Conclusion and recommendations

More than a third of children referred to a psychiatric outpatient centre were involved in bullying. This indicates a high prevalence of bullying involvement among these children. Most children of the study population were male and almost all the bullies presented with aggression. Aggressive behaviour is a referral reason that usually demands urgent attention, which might explain the high prevalence at the centre. Gender and age are also associated with aggression during childhood, which might explain this constellation of factors. The prevalence of poor academic achievement was high in all groups, showing the possibility of susceptibility to bullying involvement and psychiatric illness. An additional comorbid condition, namely ADHD with a high prevalence in the bully and victim group, indicates the necessity of a multifaceted and multidisciplinary approach. For socio-demographic characteristics, there were no statistically significant differences between the children involved and those not involved in bullying and between the bullies and the victims.

The most prominent psychiatric morbidity in this study population was conduct disorder. Although bullying and conduct disorder have distinct qualities, the study’s results motivate the inclusion of different intervention strategies in bullying prevention programmes. Bullying prevention programmes might be strengthened by incorporating the family through direct parent management training, an essential component in treating conduct disorder, and not only focusing on the child involved in bullying. The importance of screening for both victims and perpetrators of bullying involvement is also highlighted. Psychiatric intervention should also focus on the psychiatric morbidity of children involved in bullying. Early identification and intervention at primary care level might preclude the development of a psychiatric disorder, such as conduct disorder.

Community-based investigations should be conducted where bullying is most prevalent, such as in schools and neighbourhoods, to attain information on the morbidity among bullying participants. In addition to the need for prevention and early identification of bullying involvement, it is recommended that teachers, parents and other family members should be made more aware of the behavioural changes and morbidity of children involved in bullying so that they can act rapidly and effectively.
